# Evaluation of "*Candida *score" in critically ill patients: a prospective, multicenter, observational, cohort study

**DOI:** 10.1186/2110-5820-1-50

**Published:** 2011-11-30

**Authors:** Guillaume Leroy, Fabien Lambiotte, Didier Thévenin, Christian Lemaire, Erika Parmentier, Patrick Devos, Olivier Leroy

**Affiliations:** 1 Service de Réanimation Médicale et Maladies Infectieuses, Centre Hospitalier de Tourcoing, 59208 Tourcoing, France; 2 Service de réanimation, Centre Hospitalier de Maubeuge 59, France; 3 Service de réanimation, Centre Hospitalier de Lens 62, France; 4 Service de réanimation médicale, Centre Hospitalier de Roubaix 59, France; 5 Service des urgences respiratoires et de réanimation médicale, Hôpital Calmette, CHRU de Lille 59, France; 6 Département de biostatistiques, CHRU de Lille 59, France

## Abstract

**Introduction:**

Although prompt initiation of appropriate antifungal therapy is essential for the control of invasive *Candida *infections and an improvement of prognosis, early diagnosis of invasive candidiasis remains a challenge and criteria for starting empirical antifungal therapy in ICU patients are poorly defined. Some scoring systems, such as the "*Candida *score" could help physicians to differentiate patients who could benefit from early antifungal treatment from those for whom invasive candidiasis is highly improbable. This study evaluated the performance of this score in a cohort of critically ill patients.

**Methods:**

A prospective, observational, multicenter, cohort study was conducted from January 2010 to March 2011 in five intensive care units in Nord-Pas de Calais, an area from North of France. All patients exhibiting, on ICU admission or during their ICU stay, a hospital-acquired severe sepsis or septic shock could be included in this study. The data collected included patient characteristics on ICU admission and at the onset of severe sepsis or septic shock. The "Candida score" was calculated at the onset of sepsis or shock. The incidence of invasive candidiasis was determined and its relationship with the value of the "Candida score" was studied.

**Results:**

Ninety-four patients were studied. When severe sepsis or shock occurred, 44 patients had a score = 2, 29 patients had a score = 3, 17 patients had a score = 4, and 4 patients had a score = 5. Invasive candidiasis was observed in five (5.3%) patients. One patient had candidemia, three patients had peritonitis, and one patient had pleural infection. The rates of invasive candidiasis was 0% in patients with score = 2 or 3, 17.6% in patients with score = 4, and 50% in patients with score = 5 (*p *< 0.0001).

**Conclusions:**

Our results confirm that the "Candida score" is an interesting tool to differentiate among ICU patients who exhibit hospital-acquired severe sepsis or septic shock those would benefit from early antifungal treatment (score > 3) from those for whom invasive candidiasis is highly improbable (score ≤ 3).

## Introduction

Invasive *Candida *infections are the most common invasive fungal infections, accounting for 70-90% of all invasive mycoses [1]. Among the causes of nosocomial bloodstream infections, *Candida *ranks number four in the United States [2]. Incidence of candidemia varies between 0.5 and 1.4 per 10,000 patient-days in hospital and between 2 and 6.9 per 1,000 admissions in the intensive care unit (ICU) [3-7]. Invasive candidiasis is associated with high mortality, especially in ICUs [8-10]. Prompt initiation of appropriate antifungal therapy is essential for the control of invasive *Candida *infections and has been shown to reduce mortality [11-13].

Unfortunately, early diagnosis of invasive candidiasis remains a challenge, and criteria for starting empirical antifungal therapy in ICU patients are poorly defined. Recent IDSA guidelines suggest that "empirical antifungal therapy should be considered in critically ill patients with risk factors for invasive candidiasis and no other known cause of fever" [14]. Risk factors for invasive candidiasis are well identified [15]. However, these are so numerous that most ICU patients could be considered as exhibiting risk factors for invasive candidiasis. Obviously, widespread use of antifungal agents would be associated with substantially increased overall health care costs and emergence of resistance [16,17].

To both ensure appropriate and timely antifungal therapy and to avoid unnecessary use of antifungal agents, some authors have developed clinical prediction rules to identify ICU patients at high risk of candidiasis and for whom initiation of empirical antifungal therapy could be justified [18-20]. However, there are many concerns about these rules: high specificity but low sensitivity, no prospective validation, and complicated use.

In 2006, a Spanish group, using the database of the Estudio de Prevalencia de CANdidiasis project, identified four predictors of proven invasive *Candida *infection [21]. Based on these predictors, a score named "*Candida *score" was built. In 2009, the same group demonstrated a significant linear association between increasing values of the "*Candida *score" and the rate of invasive *Candida *infections [22]. Such a score could be useful to stratify the risk of proven *Candida *infection and differentiate patients who would benefit from early antifungal treatment from those for whom invasive candidiasis is highly improbable.

The goal of the present study was to evaluate, in a prospective and multicenter cohort of ICU patients developing hospital-acquired severe sepsis or septic shock, the performance of the "*Candida *score."

## Materials and methods

### Study population

This prospective observational cohort study was performed from January 2010 to March 2011 in five ICUs (Lens, Lille, Maubeuge, Roubaix, and Tourcoing) in Nord-Pas de Calais, an area from the north of France. The study protocol was submitted to the Institutional Review Board for Lille University Hospital, which gave an approval with waiver of informed consent, in agreement with French regulations concerning observational studies that do not modify existing diagnosis or therapeutic strategies.

All patients exhibiting, on ICU admission or during their ICU stay, hospital-acquired severe sepsis or septic shock could be included in this study. Exclusion criteria were community-acquired infections, age < 18 years, neutropenia defined as a total leukocyte count < 500/mm^3^, pregnant women and nursing mothers, and patients who were treated with antifungal drugs when severe sepsis or septic shock occurred.

### Study design

The primary objective of this study was to determine the incidence of invasive candidiasis among included patients and to study the relationship between the presence of invasive candidiasis and the "*Candida *score" value at the onset of severe sepsis or septic shock. The secondary objective was to analyze the antifungal therapies instituted when severe sepsis or shock occurred and to determine their relationship with the "*Candida *score" value.

### Data collection, evaluation, and definition

On ICU admission, demographic characteristics, underlying diseases, reason for ICU admission, and severity of illness were recorded by each investigator on a standardized report form. Underlying diseases included insulin-dependent diabetes mellitus, neurologic conditions, chronic obstructive pulmonary disease, chronic liver disease, chronic renal failure, congestive severe heart failure, and immunosuppression (prednisolone >0.5 mg/kg/d since more than 1 month, human immunodeficiency virus infection, cancer chemotherapy within the past 3 months, organ transplantation with ongoing immunosuppressant, bone marrow allograft or hematopoietic stem-cell transplantation, immunosuppressant, or anti-tumor necrosis factor).

According to diagnoses at the time of ICU admission, patients were classified as surgical (admission for the postoperative care of an elective or urgent surgical procedure), trauma (admission because of trauma-related acute lesions), or medical (all other patients). Severity of illness on admission in ICU was assessed by using the Simplified Acute Physiology Score (SAPS) II and the Sepsis-related Organ Failure Assessment (SOFA) score [23,24].

During the ICU stay, screening for Candida colonization was performed twice weekly by routine samples from tracheal aspirates and urine. Other samples from peripheral blood, vascular catheters, wound or drainage exudates, or other infected foci could be obtained at the discretion of the attending physician. Samples were processed by the different clinical microbiology laboratories of the participating hospitals.

When hospital-acquired severe sepsis or septic shock occurred, components of the "*Candida *score" and therapeutic data were collected. Severe sepsis and septic shock were defined according to international sepsis definition [25]. They were considered as hospital-acquired when infection was neither present nor incubating at the time of admission to the acute care setting [26]. Components of "*Candida *score" were severe sepsis, total parenteral nutrition, surgery, and multifocal *Candida *colonization. These components were defined according to criteria proposed by Leon et al. [21]. The "*Candida *score" was calculated by adding points provided by each component as proposed by Leon et al. in their second study [22]. In our cohort, the minimum value of the score was 2 points for all patients, because severe sepsis or septic shock were inclusion criteria. The total score was obtained by adding 1 point for each remaining variable, total parenteral nutrition, surgery, and multifocal *Candida *colonization. *Candida *colonization was considered multifocal when *Candida *species were isolated, at the same moment, from two or more noncontiguous foci, even if *Candida *species were different. In some patients, data about *Candida *colonization were unknown when severe sepsis or septic shock occurred (i.e., patients admitted in ICU from another ward or another hospital). Samples from tracheal aspirates or urine were obtained at admission and the final *Candida *score was only determined when cultures results were available. Finally, the decision to treat a patient with antifungal drugs during the course of this observational study was at the discretion of each attending physician. In all cases, the date of starting antifungal treatment and antifungal agents administered were recorded.

In all patients, usual risk factors for invasive *Candida *infection, not included in the "*Candida *score," such as exposition to invasive devices (endotracheal tube for mechanical ventilation, central venous catheter, implantable drug delivery system, urinary catheter), antibiotic therapy for more than 5 days within the past 2 weeks, immunosuppression, renal failure, and insulin-dependent diabetes mellitus were recorded.

Invasive candidiasis was defined according to usual criteria, such as those proposed by Leon et al. [21,22]. Briefly, candidemia was defined by at least one positive blood culture, and peritonitis was diagnosed on the basis of macroscopic findings and direct examination or positive culture for *Candida *of the peritoneal fluid collected during surgical procedure. Isolation of *Candida *species from normally sterile body fluids, such as pleural or pericardial fluid, also was considered as definite criteria of invasive candidiasis. Patient mortality at the time of ICU discharge was determined.

### Statistics

Variables were expressed as median values and ranges for numerical variables and as frequencies and percentages for categorical variables. Categorical variables were compared using the Chi-square and Fisher's exact tests. Numerical variables were compared using parametric test (Student's test) or nonparametric tests (Wilcoxson, Kruskal-Wallis) according to the sample size of the groups, the number of groups, and the normality of parameters. Statistical significance was accepted at the 5% level.

## Results

Ninety-four patients were included. Main patients' characteristics on ICU admission are summarized in Table 1. At inclusion, 73 patients exhibited septic shock and 21 had severe sepsis. The median time between ICU admission and occurrence of shock or severe sepsis was 7 (range, −1 to 37) days. Mean SOFA score was 9.9 ± 2.12.

**Table 1:** Main patients' characteristics on ICU admission

Characteristics	
Mean age, yr (±SD)	62.8 (±9.9)
Male/female ratio	71/23
Mean SAPS (±SD)	51.1 (±2.12)
Mean SOFA score (±SD)	8.32 (±1.12)
Diagnosis on ICU admission, no. (%)	
Medical	68 (72.3)
Surgical Abdominal surgery	25 (26.6) 16 (17)
Trauma	1 (1.1)
Underlying diseases, no. (%)	
Congestive severe heart failure	15 (16)
Insulin-dependent diabetes mellitus	11 (11.7)
Chronic obstructive pulmonary disease	26 (27.7)
Chronic liver disease	12 (12.8)
Chronic neurologic disease	10 (10.6)
Chronic renal failure	11 (11.7)
Immunosuppression	9 (9.6)

When severe sepsis or shock occurred, 27 patients had a total parenteral nutrition, 31 had undergone a surgical procedure immediately before or during ICU stay, and 17 had a multifocal *Candida *colonization. *Candida *species were found in urine (n = 17) and tracheal aspirates (n = 17) cultures. Thirty-seven *Candida *isolates were identified: *C. albicans *(n = 30), *C. tropicalis *(n = 3), *C. parapsilosis *(n = 2), and other species (n = 2). Calculated and distribution of "*Candida *scores" was as follows: 44 patients with no risk factor had a score = 2; 29 patients with a lone risk factor (total parenteral nutrition n = 8, surgery n = 10, and multifocal *Candida *colonization n = 11) had a score = 3; 17 patients with two risk factors (total parenteral nutrition n = 15, surgery n = 17, and multifocal *Candida *colonization n = 2) had a score = 4. Finally, four patients had all risk factors and a score = 5.

Numerous other risk factors for invasive *Candida *infection were present and are reported in Table 2, broken by "*Candida *score" value. Among the 94 patients, severe sepsis or septic shock were due to or associated with invasive candidiasis in 5 (5.3%) patients. One patient had *C. parapsilosis *candidemia, three patients had *C. albicans *peritonitis, and one patient had *C. tropicalis *pleural infection. The patient with *C. parapsilosis *candidemia had *C. albicans*-positive tracheal aspirates cultures and *C. parapsilosis-*positive urine cultures. Among the three patients with peritonitis, only one had multifocal *Candida *colonization with *C. albicans-*positive tracheal aspirates and urine cultures. The patient with *C. tropicalis *pleural infection had *C. albicans*-positive tracheal aspirates and urine cultures.

**Table 2:** Risk factors for invasive candidiasis, according to the value of "*Candida *score"

Risk factors	*Candida *score = 2 (n = 44)	*Candida *score = 3 (n = 29)	*Candida *score = 4 (n = 17)	*Candida *score = 5 (n = 4)
Severe sepsis or septic shock	44	29	17	4
Total parenteral nutrition	0	8	15	4
Surgery	0	10	17	4
Multifocal *Candida *colonization	0	11	2	4
Invasive mechanical ventilation	30	23	11	2
Central venous catheter	39	27	15	4
Urinary catheter	42	27	17	4
Antibiotherapy > 5 days within the past 2 weeks	39	25	14	4
Renal replacement therapy	8	10	4	1
Insulin-dependent diabetes mellitus	7	4	0	0
Immunosuppression	4	3	2	0

The rates of invasive candidiasis according to the "*Candida *score" was 0% in the 44 patients with score = 2 and the 29 patients with score = 3, 17.6% (n = 3) in the 17 patients with score = 4, and 50% (n = 2) in the 4 patients with score = 5. The association between increasing values of the "*Candida *score" and the rate of invasive candidiasis is statistically significant (*p *< 0.0001). In patients with a "Candida score" ≤3, no invasive candidiasis was observed. The positive and negative predictive values of this cutoff value of the "Candida score" were 23.8% and 100%, respectively. Among the 68 patients with medical diagnosis on ICU admission, 4 had a "*Candida *score" >3 and 1 of 4 exhibited an invasive candidiasis. Among the 25 patients with a surgical diagnosis on ICU admission, 17 had a "*Candida *score" >3 and 4 of 17 had an invasive candidiasis. Although the number of patients with a "*Candida *score" >3 was higher in surgical than medical patients (*p *< 0.0001), the rate of invasive candidiasis was similar for surgical and medical patients with a "*Candida *score" >3 (4/17 vs. 1/4; *p *= 1). When severe sepsis or septic shock occurred, 19 (20%) patients received empiric antifungal treatment with fluconazole in 12 cases and caspofungin in 7 cases. Empiric antifungal treatment was initiated in 1 of 44 (2.3%) patients with a "*Candida *score" = 2, 8 of 29 (27.6%) patients with a "*Candida *score" = 3, 7 of 17 (41.2%) patients with a "*Candida *score" = 4, and 3 of 4 (75%) patients with a "*Candida *score" = 5. All five patients with invasive candidiasis received, when severe sepsis or septic shock occurred, empirical antifungal therapy.

Forty-six (49%) patients died during their ICU stay. Among the 68 patients with a medical diagnosis on ICU admission, 32 (47%) died, whereas among the 25 patients with a surgical diagnosis on ICU admission, 14 (56%) died (*p *= 0.45). Mortality rates according to the values of the "*Candida *score" and the empiric initiation of antifungal treatment are reported in Table 3. No statistically significant difference was observed. Among the 73 patients with a "*Candida *score" ≤3, 33 (45%) died, whereas among the 21 patients with a score >3, 13 (62%) died (*p *= 0.17). An empiric antifungal treatment was instituted in 19 patients, and 10 (53%) of them died. Thirty-six (48%) of the 75 patients who did not receive empiric antifungal treatment died. Such a difference was not statistically significant (*p *= 0.72). Finally, among the 21 patients with a "*Candida *score" >3, mortality was not different for patients who received or did not receive empiric antifungal treatment (6/10 vs. 7/11; *p *= 1).

**Table 3:** Severity scores and mortality, according to the value of "Candida score"

	*Candida *score = 2 (n = 44)	*Candida *score = 3 (n = 29)	*Candida *score = 4 (n = 17)	*Candida *score = 5 (n = 4)	*p*
Mean SAPS ± SD	51.61 ± 16.79	49.72 ± 15.93	52.18 ± 21.84	52 ± 8.04	0.981
Mean SOFA score ± SD	8.8 ± 3.3	7.41 ± 3.27	8.59 ± 4.71	8.5 ± 1.73	0.583
Overall mortality	22 (50%)	11 (38%)	11 (64.7%)	2 (50%)	0.37

Mortality according to the initiation of an empiric antifungal treatment					

	*Candida *score = 2	*Candida *score = 3	*Candida *score = 4	*Candida *score = 5	*p*

Yes	1/1	3/8	4/7	2/3	0.59
No	21/43	8/21	7/10	0/1	0.3

## Discussion

This prospective cohort study confirms that the "*Candida *score" is an interesting tool to differentiate, among ICU patients with hospital-acquired severe sepsis or septic shock, those who would benefit from early antifungal treatment (score > 3) from those for whom invasive candidiasis is highly improbable (score ≤ 3).

For patients who develop, during ICU stay, severe sepsis or septic shock, it has been demonstrated a few decades ago that prompt and adequate antimicrobial treatment is associated with prognosis improvement. Moreover, it was demonstrated that bloodstream infections due to *Candida *species were independently associated with administration of inadequate antimicrobial treatment [27]. These results led physicians to question the interest of adding an antifungal agent to the empirical antibiotic regimen administered to patients with severe sepsis or septic shock. However, criteria for starting empirical antifungal therapy in ICU patients are poorly defined and recent IDSA guidelines suggesting that "empirical antifungal therapy should be considered in critically ill patients with risk factors for invasive candidiasis and no other known cause of fever" could lead to an overuse of antifungal agents. The "*Candida *score" could overcome the dilemma between overuse, associated with increased health costs and development of resistance, and underuse, associated with prognosis impairment of critically ill patients.

In 2006, Leon et al. reported a prospective, cohort, observational, multicenter study of 1,669 adult ICU patients admitted to 73 medical-surgical Spanish ICUs between May 1998 and January 1999 [21]. They identified four independent factors associated with a greater risk for proven invasive *Candida *infection. A bedside scoring system called "*Candida *score" was built as follows: *Candida *score = 0.908 × (total parenteral nutrition) + 0.997 × (surgery) + 1.112 × (multifocal *Candida *species colonization) + 2.038 × (severe sepsis). All variables were coded 1 if present and 0 if absent. According to receiver operating characteristics (ROC) curve and area under the ROC curve, a score > 2.5 accurately identified patients with the greater risk of proven invasive *Candida *infection (sensitivity 81%, specificity 74%). In 2009, Leon et al. prospectively studied 1,107 adult ICU patients admitted between April 2006 and June 2007 in 36 medical-surgical ICU in Spain, France, and Argentina [22]. They used a rounded "*Candida *score" calculated as follows (all variables were coded 0 if absent and 1 if present): 1 × (total parenteral nutrition) + 1 × (surgery) + 1 × (multifocal *Candida *colonization) + 2 × (severe sepsis). They demonstrated a linear and significant association between increasing values of the "*Candida *score" and the rate of invasive candidiasis. The incidence rate was 2.3, 8.5, 16.8, and 23.6 when the score was <3, 3, 4, and 5, respectively. They concluded that invasive candidiasis is highly improbable in patients with "*Candida *score" <3.

Our results are in accordance with Leon's data. The incidence rates of invasive candidiasis were 0%, 0%, 17.6%, and 50% in patients with scores equal to 2, 3, 4, and 5, respectively. A linear and significant association between increasing values of the "*Candida *score" and the rate of invasive candidiasis was observed, and no invasive candidiasis occurred in patients with scores <3. Moreover, no invasive candidiasis was observed in patients with a "*Candida *score" = 3. Interestingly, we also can underline that the performances of this score are similar in medical and surgical patients. Surgical patients exhibit more often a "*Candida *score" >3 than medical patients, but the rate of invasive candidiasis is similar in surgical and medical patients with a "*Candida *score" >3.

Therapeutic decisions of intensivists in our group could be discussed. Decision to initiate antifungal drugs during this observational study was at the discretion of each attending physician. Nevertheless, we observed a relationship between initiation of antifungal agents and value of the "*Candida *score." Frequency of antifungal empirical therapy were 2.3%, 27.6%, 41.2%, and 75% in patients with scores equal to 2, 3, 4, and 5, respectively. In our opinion, this is rather satisfying because most patients with highly improbable invasive candidiasis did not receive antifungal drugs, whereas early empirical antifungal treatment was initiated in most patients who would benefit from it. However, the fact that only a quarter of patients with score = 3 received an empiric antifungal treatment merits some comments. In our series, all included patients exhibited severe sepsis or septic shock and, therefore, had a "*Candida *score" always at least equal to 2. Initiation of empirical antifungal treatment to all patients with another risk factor (surgery or total parenteral nutrition or multifocal *Candida *colonization) probably seemed to be excessive to most intensivists of our group. The absence of invasive candidiasis, among the 29 patients with a "*Candida *score" = 3, retrospectively confirm the appropriateness of their decision.

This study has numerous limitations. First, the number of included patients is low compared with the number of patients studied by Leon and his group [21,22]. The inclusion of patients with severe sepsis or septic shock rather than all ICU admitted patients, and the low number of ICU participating to this cohort study explain this fact. Second, only five invasive candidiasis and, among them, only one candidemia were observed in this cohort. The low number of invasive candidiasis can be explained, first, with some exclusion criteria such as neutropenia, and, second, with the setting of the ICU; four of them were in a secondary care hospital and only one in a university hospital. Third, in some patients, the "*Candida *score" could not be calculated when sepsis or shock occurred, because some mycological data were lacking. However, this only reflects the shortcomings of a real-life study and represents one limit to the practical use of the score.

## Conclusions

Our study confirms the clinical relevance of the "*Candida *score" in patients with hospital-acquired severe sepsis or septic shock. This score can differentiate patients who would benefit from early antifungal treatment from those for whom invasive candidiasis is highly improbable.

## Competing interests

The authors declare that they have no competing interests.

## Authors' contributions

GL contributed to the design of the study, collected the data, and wrote the manuscript. FL, DT, CL, and EK collected the data. PD performed the statistical analysis. OL contributed to the design of the study and wrote the manuscript. All the authors read and approved the final manuscript.
